# Neuropsychiatric Symptoms in Patients with Early‐onset Dementia from a Memory Clinic in India

**DOI:** 10.1002/alz.088117

**Published:** 2025-01-03

**Authors:** Atri Chatterjee, Sanghamitra Laskar, Ankur Arvind Abhinav, Sonali Aggarwal, Aparna Kathait

**Affiliations:** ^1^ Vardhman Mahavir Medical College & Safdarjung Hospital, New Delhi, Delhi India; ^2^ Vardhman Mahavir Medical College & Safdarjung Hospital, New Delhi, New Delhi India

## Abstract

**Background:**

Neuropsychiatric symptoms (NPS) are common in patients who develop dementia before the age of 65 years, defined as early‐onset dementia (EoD). NPS are a major source of morbidity and caregiver distress in patients living with EoD. The prevalence, severity and types of NPS in different populations are unclear. Therefore, we investigated the frequency and characteristics of NPS in EoD patients attending a memory clinic in India.

**Method:**

We recruited participants with a clinical diagnosis of EoD from a subspecialty cognitive neurology clinic. NPS were assessed using Neuropsychiatric Inventory‐Questionnaire (NPI‐Q). In addition, we collected information regarding age at onset, gender, diagnosis and duration of disease. We analyzed the frequency and severity of NPS among the participants present at the baseline assessment.

**Result:**

We report data from 31 patients diagnosed with EoD. The average age at baseline assessment was 57.1 years (SD ± 7.9) and the mean age at onset of symptoms was 54 years (SD ± 6.6). 61.3% patients were male. 38.7% patients were diagnosed with vascular cognitive impairment; 32.3% and 9.7% patients were diagnosed with Alzheimer’s disease and frontotemporal dementia respectively. 96.8% patients reported at least one NPS with a mean NPI‐Q total score of 13.6 (SD ± 15.2). Irritability stood as the most frequent symptom (61.3%), followed by agitation (58.1%) and apathy (45.2%). Notably, 12.9% of patients reported psychotic symptoms at baseline.

**Conclusion:**

NPS were nearly ubiquitous in this Indian EoD cohort at baseline. Irritability, agitation, and apathy, associated with significant caregiver burden, emerged as the dominant NPS. Further research is crucial to explore the efficacy of pharmacotherapy and culturally relevant non‐pharmacological interventions for managing NPS in diverse populations.

